# Microbial community structure and functional potential of lava-formed Gotjawal soils in Jeju, Korea

**DOI:** 10.1371/journal.pone.0204761

**Published:** 2018-10-12

**Authors:** Jong-Shik Kim, Dae-Shin Kim, Keun Chul Lee, Jung-Sook Lee, Gary M. King, Sanghoon Kang

**Affiliations:** 1 Gyeongbuk Institute for Marine Bioindustry, Uljin, Republic of Korea; 2 World Heritage and Mt. Hallasan Research Institute, Jeju Special Self-Governing Province, Republic of Korea; 3 Korean Collection for Type Cultures, Korea Research Institute of Bioscience and Biotechnology, Jeongup, Republic of Korea; 4 Biological Sciences, Louisiana State University, Baton Rouge, LA, United States of America; 5 Department of Biology, Baylor University, Waco, TX, United States of America; Free University of Bozen/Bolzano, ITALY

## Abstract

The Gotjawal areas of Jeju Island, Korea, are comprised of unmanaged forests growing on volcanic soils. They support unique assemblages of vascular plants from both northern and southern hemispheres, but are threatened by human disturbance. The health and ecosystem function of these assemblages likely depends in part on the diversity and community structure of soil microbial communities, about which little is known. To assess the diversity of Gotjawal soil microbial communities, twenty samples were collected in November 2010 from 4 representatives of Gotjawal forests. While soil properties and microbial communities measured by 16S rRNA gene sequence data were marginally distinct among sites by PERMANOVA (*p =* 0.017–0.191), GeoChip data showed significant differences among sites (*p* <0.006). Gene composition overall, and the composition of 3 functional gene categories had similar structures themselves and similar associations with environmental factors. Among these communities, phosphorous cycling genes exhibited the most distinct patterns. 16S rRNA gene sequence data resulted in a mean 777 operational taxonomic units (OTUs), which included the following major phyla: *Proteobacteria* (27.9%), *Actinobacteria* (17.7%), *Verrucomicrobia* (14.3%), *Acidobacteria* (9.6%), *Planctomycetes* (9.8%), *Bacteroidetes* (8.9%), and *Chloroflexi* (2.2%). Indicator species analysis (ISA) was used to determine the taxa with high indicator value, which represented the following: uncultured *Chlamydiaceae*, *Caulobacter*, uncultured *Sinobacteraceae*, *Paenibacillus*, *Arenimonas*, *Clostridium* sensu.stricto, uncultured *Burkholderiales* incertae sedis, and *Nocardioides* in Aewol (AW), *Aquicella*, uncultured *Planctomycetia*, and *Aciditerrimonas* in Gujwa-Seongsan (GS), uncultured *Acidobacteria* Gp1, and *Hamadaea* in Hankyeong-Andeok (HA), and *Bosea*, *Haliea*, and *Telmatocola* in Jocheon-Hamdeok (JH) Gotjawal. Collectively, these results demonstrated the uniqueness of microbial communities within each Gotjawal region, likely reflecting different patterns of soil, plant assemblages and microclimates.

## Introduction

On Jeju Island, Korea, the term “Gotjawal” refers to a unique natural unmanaged forest that grows on widely distributed lava-based formations characterized by dense stands of trees and shrub undergrowth[1]. The Gotjawal provides numerous ecosystems services, including groundwater purification[2], that have supported human occupation historically. At present, some areas of the Gotjawal are protected, but about 50% of the original forests have been replaced by secondary growth due to losses from deforestation, agricultural land use, and urbanization[3]. Gotjawal soils are considered highly fertile due to their high organic matter and low mineral content, but relatively little is known about their microbiology[2, 4]. Previous cultivation-based studies of these soils have identified several new genera and species [2, 4–8], but more comprehensive analyses are needed to understand patterns of microbial community structure and function, and interactions with Gotjawal plant assemblages.

Studies on existing volcanic systems have focused on microbial distribution and function in relation to lava flows, their ages and plant or ecosystem development (for review see[2]). The microbial diversity of volcanic deposits is poorly characterized, but several studies have shown that lava weathering partially regulates rates of nitrogen fixation in young Mauna Loa ecosystems (Hawai`i) [9], and is accompanied by changes in carbon monoxide oxidizer diversity during succession on recent volcanic deposits at Kilauea Volcano, Hawai`i[10].

We report here the structure and functional potential of Gotjawal microbial communities analyzed using GeoChip 4.0 and the Illumina MiSeq platform for 16S rRNA gene sequences. First, we set out to elucidate the diversity and community structure of microbes in Gotjawal soils, along with determining their functional potential. Second, we identified the major environmental factors that shape the microbial community structure in Gotjawal soils. The results suggest that Gotjawal forests with distinct biotic and physical characteristics support distinct microbial communities.

## Materials and methods

### Sampling sites

**Fig 1:**
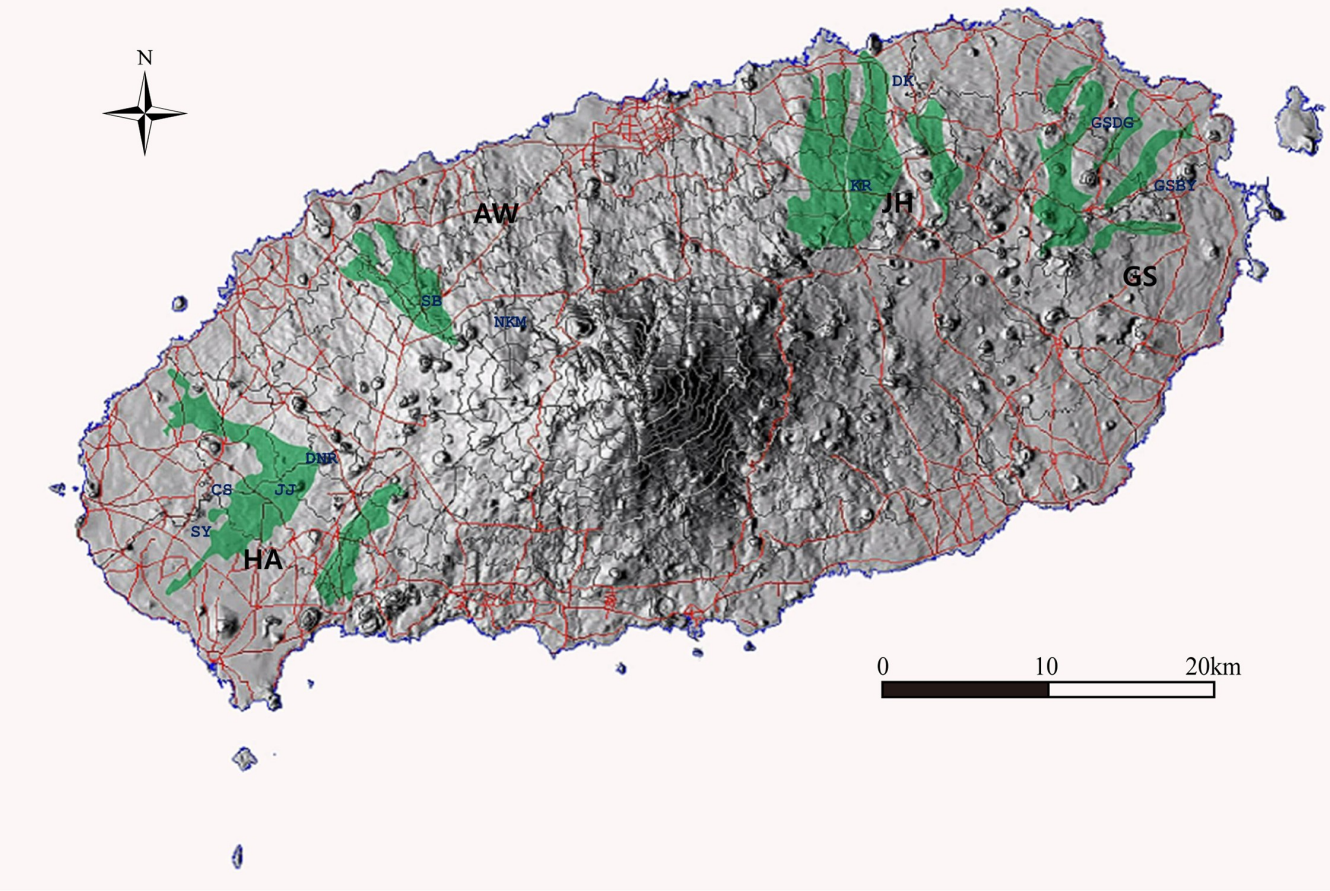
Map of Gotjawal forest sampling sites. The map is created using ArcGIS 10.3 (http://www.esri.com/arcgis/about.arcgis) and Surfer 8.0 software (http://www.goldensoftware.com/products/surfer).

Gotjawal forests are located along the east-west axis of Jeju Island, Korea. Most forests occur in inland areas at altitudes of 200 to 400 m. Gotjawal forests tend to form a zone between inhabited coastal areas and mountainous regions used for grazing livestock. Four major Gotjawal forests are spread across Jeju Island, separated by Mount Hallasan (1,950 m) in the middle of the island (Fig 1). These 4 forests have been preserved well (S1 and S2 Tables and S1 Fig).

### Soil collection and DNA extraction

Soil was sampled from all sites in November 2010. No specific permissions were required for sampling the soils, and the activities did not involve endangered or protected species. Soil samples from each sampling location were collected from behind or between lava and tree roots as one sample with mixed 6 subsamples. Each two samples were collected at distances of 10 m to 30 m in the field. Samples were collected aseptically using ethanol-disinfected shovels and were placed in clean, sealable plastic bags. Sampled soils were sieved using 2-mm mesh sieves autoclaved on the day of sampling. The samples were first stored in a 4°C cooler during transfer to a laboratory (Uljin, South Korea), then stored at 4°C for a week prior to shipment overnight to Glomics Inc. (Norman, OK, USA). DNA was extracted from approximately 0.25 g Gotjawal soil using the Power Soil DNA Isolation Kit (MoBio Laboratories, Carlsbad, CA, USA) for GeoChip and using a FastDNA SPIN kit for soil (QBiogene Inc., Vista, CA) for 16S rRNA gene sequencing using Illumina MiSeq. Total microbial DNA was quantified with Quant-iT PicoGreen (Invitrogen, Carlsbad, CA, USA) according to the manufacturer's instructions.

### Soil analyses

Soil physical-chemical properties were measured at the National Instrumentation Center for Environmental Management (NICEM, Seoul, South Korea) based on standard SSSA (Soil Science Society of America) protocols (Table 1). Variables analyzed included pH, EC electrical conductivity, OM (organic matter content), TOC (total organic carbon content), T-N (total nitrogen content), NH_4_^+^, NO_3_^-^, CEC (cation exchange capacity, Mg, Na, Ca, K), P_2_O_5_, clay, silt and sand.

**Table 1:** Chemical properties of Gotjawal soil samples.

Gotjawal		HACS-1	HACS-2	HADNR-1	HADNR-2	HASY-5	HASY-6	HAJJ-1	HAJJ-2	AWNKM-1	AWNKM-2	AWSB-1	AWSB-2	JHKR-1	JHKR-2	JHDK-1	JHDK-2	GSBY-1	GSBY-2	GSDG-1	GSDG-2
**pH**		5.5	4.0	5.3	6.2	4.2	4.5	5.3	4.6	5.0	5.3	5.1	5.5	4.5	4.5	5.2	4.5	5.5	5.6	5.4	5.7
**EC**	dS/m	2.69	2.62	5.06	3.83	1.67	2.33	2.05	1.88	7.41	8.01	3.61	4.03	2.80	4.09	1.87	1.69	3.72	3.15	3.26	3.59
**OM**	%	44	46	50	45	25	30	43	34	66	66	36	69	33	35	29	31	44	43	42	46
**TOC**	%	25.25	26.57	29.24	25.91	14.61	17.28	25.15	19.85	38.24	38.30	21.10	40.19	19.17	20.14	16.83	18.04	25.44	25.09	24.35	26.79
**TN**	%	1.71	2.31	2.39	2.15	0.79	1.30	2.32	1.52	2.46	2.64	1.71	2.83	1.49	1.83	1.36	1.33	2.03	1.97	2.60	1.98
**NH**_**4**_**(+)**	(mg/kg)	72.860	70.595	113.961	128.080	174.471	93.790	110.935	115.978	150.267	124.046	259.186	164.386	255.151	288.432	91.774	80.680	156.318	154.301	168.420	155.309
**NO**_**3**_**(-)**	(mg/kg)	132	573.17	330.004	434.217	204.950	149.371	243.160	166.740	500.216	406.428	389.059	628.746	191.057	409.900	114.651	66.017	357.793	361.270	382.110	455.058
**CEC**	(cmol/kg)	66.05	75.19	95.88	86.28	55.74	62.88	67.74	55.12	104.35	106.23	76.95	101.02	63.62	63.43	59.31	56.80	94.51	94.51	72.29	74.28
**Exch. Cation (mg/kg)**	Ca	3476.3	1100.56	8569.35	8320.66	97.53	721.33	5616.33	872.46	8172.99	9914.45	3793.39	8897.49	729.57	1745.03	2557.95	568.87	5997.16	5859.38	4983.58	6283.64
	Mg	868.86	400.75	1723.10	1273.95	299.07	196.04	1089.31	557.90	1511.22	1688.29	646.63	1446.84	167.56	316.82	813.11	337.37	866.75	838.50	618.30	866.29
	K	179.43	139.79	500.60	418.60	96.60	94.12	263.66	238.21	601.34	511.97	369.26	446.59	232.30	258.42	184.85	203.06	315.88	284.01	265.84	341.37
	Na	82.63	39.04	54.63	71.58	43.60	18.27	65.83	55.74	80.21	75.87	23.17	63.81	30.26	35.76	84.13	87.02	44.38	44.46	58.06	56.39
**Aval. P**_**2**_**O**_**5**_		79.98	70.13	203.54	99.59	33.47	112.47	135.88	57.11	250.72	214.64	84.17	119.55	37.86	59.03	49.09	61.15	91.39	97.50	109.35	68.41
**soil texture**		loam	clay	clay loam	sandy loam	silt loam	silt clay loam	clay loam	clay loam	clay	sandy clay	sandy clay loam	silty clay	sandy loam	sandy loam	loam	loam	sandy loam	sandy loam	sandy clay loam	sandy loam
**sand**		38.87	11.47	32.26	66.82	1.99	2.78	29.62	20.07	39.57	51.87	64.19	3.65	66.97	56.11	46.92	32.46	61.13	62.13	61.52	74.54
**silt**	%	35.28	37.79	36.22	20.89	77.90	69.51	42.73	50.41	19.84	11.19	9.93	53.26	19.66	25.80	33.42	45.81	23.16	24.60	17.99	14.64
**clay**		25.86	50.74	31.52	12.29	20.11	27.71	27.65	29.52	40.59	36.94	25.87	43.10	13.37	18.09	19.66	21.74	15.71	13.26	20.48	10.81
**Heavy Metal**	**(mg/kg)**																				
**Fe**		#####	12421.00	4946.50	14637.67	38434.33	19017.67	14394.33	23287.67	6794.75	4482.25	15551.00	5846.50	35967.67	22397.67	28677.7	25001.00	19717.67	23231.00	24461.00	19381.00
**Mn**		438.60	200.60	221.77	540.60	236.67	209.60	634.60	424.27	415.85	534.40	869.60	427.93	429.93	330.20	440.93	482.27	763.60	935.93	790.60	689.93
**Si**		241.03	235.97	172.70	361.90	217.03	455.57	331.23	274.73	378.25	441.35	674.90	179.33	162.00	657.23	248.23	350.57	451.90	1325.23	603.23	629.57
**Al**		#####	4801.67	3002.00	10488.33	14181.67	8758.33	9341.67	14151.67	3928.50	2887.00	6645.00	3258.67	24265.00	17935.00	14381.7	17355.00	15341.67	18751.67	13348.33	15198.33
**V**		36.73	28.07	9.03	28.17	91.47	44.47	27.53	48.67	12.45	7.70	30.27	10.90	66.00	35.17	64.57	49.53	39.70	47.53	53.80	41.50
**Cd**		1.23	0.87	0.60	1.23	2.30	1.13	1.27	1.50	1.05	1.15	1.37	1.13	2.57	1.77	1.93	1.80	1.63	1.87	2.00	1.57
**Cu**		10.77	8.97	11.60	19.33	4.37	10.13	15.27	13.73	19.20	21.75	17.63	18.27	20.47	15.13	11.97	15.37	23.73	29.20	26.27	19.07
**Pb**		16.03	19.27	16.30	37.43	21.30	19.87	26.13	19.83	40.90	37.75	78.43	36.83	23.37	25.30	23.93	22.17	29.43	32.00	32.37	22.83
**Ni**		12.50	7.80	7.13	17.27	13.60	8.17	12.80	20.50	8.85	9.10	10.00	7.87	20.70	13.93	12.33	18.03	16.83	20.23	19.90	24.77

### GeoChip hybridization and data preprocessing

GeoChip hybridization and data pre-processing were carried out by Glomics Inc. using their data processing pipeline (http://ieg.ou.edu/microarray/). Briefly, community DNA from soil samples was randomly amplified then labeled with Cy3 fluorescent dye before hybridization with randomly placed Cy5-labeled common oligo reference standard (CORS) target and Cy3-labeled alignment oligo (Roche NimbleGen, Madison, WI, USA). The hybridized array was scanned with a NimbleGen MS200 Microarray Scanner and quantified with NimbleScan software with an appropriate grid file. Data pre-processing was carried out using the Microarray Data Management (MGM) system on the Institute for Environmental Genomics (IEG) website (http://ieg.ou.edu/microarray). The probes were considered positive if the signal-to-noise ratio (SNR) was > 2.0 and the coefficient of variability of the background was < 0.8, Signal intensity for each probe were normalized by the mean signals from all spiked CORS probes

### 16S rRNA gene sequencing and sequence processing

Briefly, the V3-V4 region of bacterial 16S rRNA gene amplicons were generated using the forward primer (337F, 5' CCTACGGGNGGCWGCAG) and reverse primer (805R, 5'GACTACHVGGGTATCTAATCC)[11]. Each sequenced sample was prepared according to the Illumina 16S Metagenomic Sequencing Library protocols. The quantification of DNA and the DNA quality was measured by PicoGreen (Invitrogen) and Nanodrop (Thermo Scientific). Input gDNA (10ng) was PCR amplified. The final purified product was then quantified using qPCR according to the qPCR Quantification Protocol Guide and qualified using the Agilent Tapestation D1000. These libraries were sequenced by multiplexed paired-end sequencing on the Illumina MiSeq (Illumina, San Diego, CA, USA) platform v3 technology (2 × 300 bp, paired-end) by Macrogen Inc. (Seoul, Korea).

After contigs were formed from the paired-end sequences by FLASH (v.1.2.11)[12], additional sequence processing was done using CD-Hit-OTU[13]. Short sequences of less than 40% in library and sequencing errors (Phred score < 30%), homopolymer and ambiguous bases were removed. Chimeric sequences were identified and removed using (USEARCH)[14]. The remaining sequences were used to form Operational Taxonomic Units (OTU, cluster cutoff value, 97%). Singletons and doubletons were not used for downstream analyses. Each representative OTU sequence were compared against the RDP database (release 11) the taxonomy was assessed using UCLUST (v.1.2.22)[14]. Species diversity and evenness were assessed by calculating Shannon index, Simpson index, Chao1, and Good’s coverage. In addition, the most characteristic species of the microbial community in four Gotjawal areas were identified through the indicator value using the indval function of labdsv package in the program R (www.r-project.org). The sequence data were deposited in the NCBI Sequence Read Archive (SRA) under the BioSample accession numbers SAMN06049757 to SAMN06049776.

### Statistical analysis

Nutrient cycle genes (C, N, P, and S cycles) were selected from the GeoChip data for further analysis in addition to all functional genes. The OTU table from 16S rRNA gene sequences was used for establish the taxonomy of Gotjawal microbial communities. Non-metric multidimensional scaling (NMDS) was used for unconstrained ordination of microbial communities. The NMDS results were quantitatively evaluated with analysis of similarity (ANOSIM) and permutational multivariate analysis of variance (PERMANOVA). NMDS configurations were compared using the Procrustes test[15]. Soil characteristics were fitted using vector fitting[16]. Hierarchical cluster analysis (HCA) and K-means cluster analysis were performed to classify the samples from the 4 Gotjawal areas[17]. HCA was carried out with Pearson coefficient without centering for distance measurement and the complete linkage agglomerative cluster algorithm[18], because it has been proposed to be the most legitimate cluster algorithm for microarray data in hierarchical cluster analysis[19].

The data structure was investigated by comparing the results from the redundancy analysis (RDA) and canonical correspondence analysis (CCA) for the GeoChip dataset, 16S rRNA sequence dataset, and soil characteristics (16 variables). The results clearly supported a unimodal data structure; thus, CCA was used to link microbial communities and soil characteristics. The initial results were very noisy, possibly due to the complex structure of the high variability in soil characteristics. Thus, subsets of soil characteristics were chosen by an iterative procedure of CCA modeling, based on VIF (variance inflation factor) and testing the significance of environmental constraints beginning with the full model. Specifically, variables with the highest VIF were sequentially removed until all variables became non-redundant, while the significance of soil characteristic constraints was controlled as *p* < 0.05. To understand the effect of geographic distances among samples, Mantel test and multivariate correlogram analysis were performed. Mantel test and partial Mantel test[17] were also used for additional gradient analysis.

All of the analyses were performed in R v. 3.0.2, with packages vegan v. 2.2–1 and ecodist v. 1.2.9[20].

## Results

### Soil characteristics

Soil physical and chemical properties varied among the sites (Table 1). The characteristics of GS sites were distinct from the others, whereas the JH and AW sites clustered together, overlapping minimally with GS and HA (S2A Fig). In contrast, an HA site (HASY1) differed distinctly on the non-metric multidimensional scaling (NMDS) ordination field. Similar patterns were observed in hierarchical cluster analysis (HCA), with all first-level clusters containing samples from the various sites (S2B Fig). Composite soil characteristics were marginally significantly classified for the 4 regions by analysis of similarity (Table 1). However, both Mantel test with geographic distances (*r*_M_ = 0.473, *p* = 0.002) and Procrustes test with NMDS ordination with geographic locations (*t* = 0.554, *p* < 0.001) were highly significant, indicating the soil characteristics were spatially autocorrelated.

### Microbial communities

**Fig 2:**
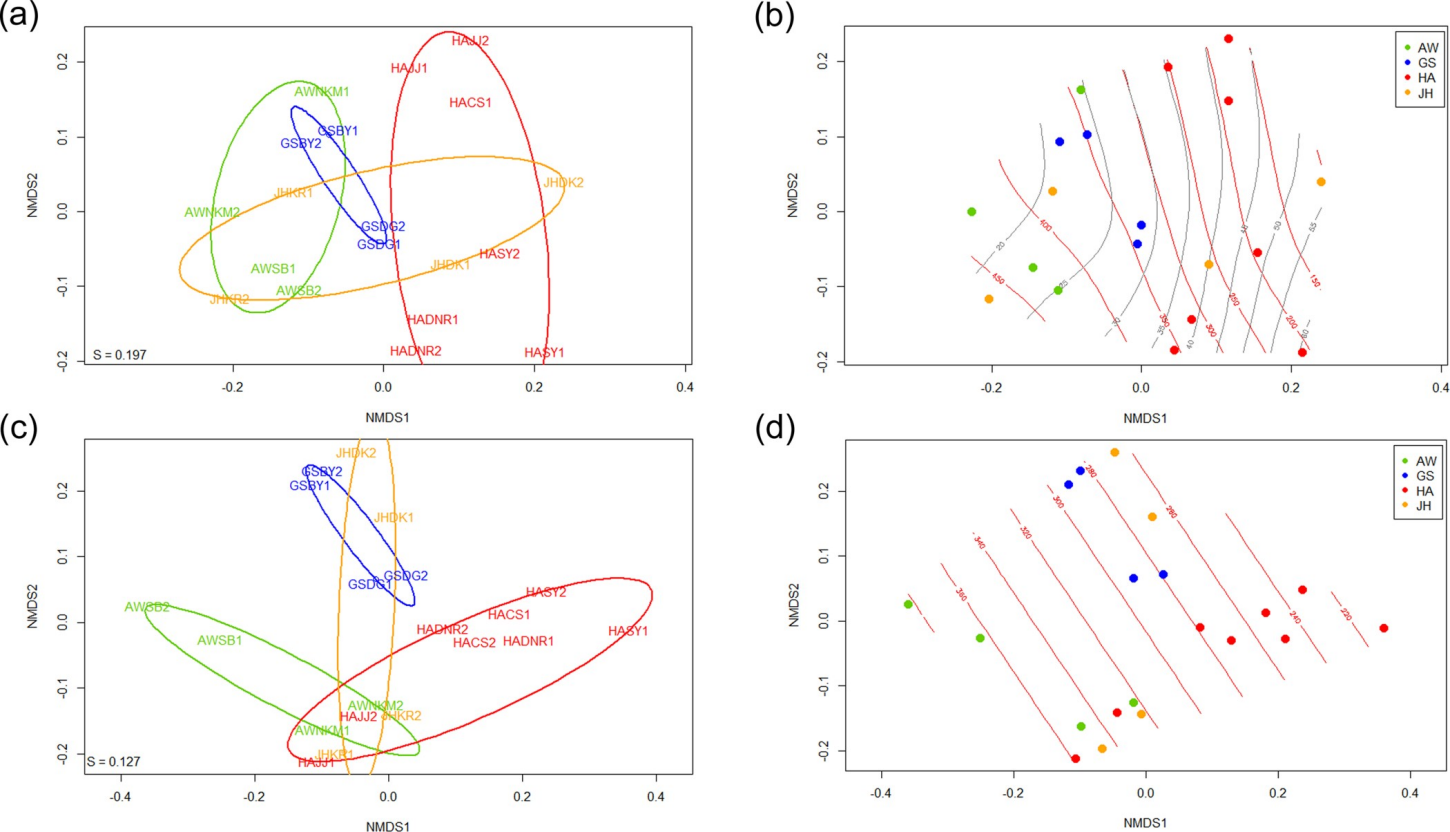
Exploratory analysis of microbial communities by unconstrained ordination plots using Non-metric Multidimensional Scaling (NMDS) with 16S rRNA gene sequence (a & b) and GeoChip functional gene hybridization data (c & d) with ellipses determined by the standard deviation of point scores (a & c) and with gradients of two most important soil variables by GAM fitting (b-nitrate (red) and %silt (grey) & d- K-exchange capacity).

The diversity of 16S rRNA gene and soil microbial functional genes by both Shannon index (*H*’) and effective diversity of Shannon index significantly among the 4 Gotjawal regions (S3 Fig). Soil microbial communities defined by functional genes clustered for all 4 regions, based on analysis of similarity (ANOSIM, soil (R = 0.118, *p* = 0.117), 16S (R = 0.091, *p* = 0.191), GeoChip (R = 0.318–0.410, *p* ≤ 0.005) and permutational multivariate analysis of variance (PERMANOVA, soil (F = 3.411, *p* = 0.026, 16S (F = 1.628, *p* = 0.017), GeoChip (F = 2.476–2.753, *p* < 0.001)) (Table 2). However, the 4 regions showed no clear delineation with respect to 16S rRNA gene sequences when using either non-metric multidimensional scaling (NMDS) ordination (S4 Fig) or hierarchical cluster analysis (HCA) (S5 Fig). Interestingly, while there was significant distinction between microbial community structures represented by 16S rRNA genes and microbial functional genes (Fig 2) (*R* = 0.302, *p* = 0.308), microbial community structures represented by nutrient cycle genes were very similar among each other and with all together (S4 Fig) (*R* > 0.98, *p* < 0.001). Overall, the GS Gotjawal region microbial communities were well clustered consistently, and other Gotjawal regions contained fairly diverse soil microbial communities.

**Table 2:** PERMANOVA and analysis of similarity (ANOSIM) results between 4 forests.

		Soil	16S*	GeoChip All	GeoChip C	GeoChip N	GeoChip P	GeoChip S
PERMANOVA	*F*	3.411	1.628	2.668	2.703	2.688	2.753	2.476
	*P*	**0.026**	**0.017**	**<0.001**	**<0.001**	**<0.001**	**<0.001**	**0.006**
ANOSIM	*R*	0.118	0.091	0.344	0.347	0.335	0.410	0.318
	*P*	0.117	0.191	**0.003**	**0.003**	**0.005**	**0.002**	**0.002**

* Without HACS2, results become more significant (F = 1.657, P = 0.012; R = 0.184, P = 0.051).

All statistical analyses distinguished P cycling genes from other nutrient cycling genes in the microbial communities. The NMDS ordination configurations of all nutrient cycling genes and 3 other gene (N, P, S) were virtually identical, because the *p* values from Procrustes test exceeded 0.992. In comparison, P cycling genes were consistently less similar than the other gene categories. HCA results showed that the GSBY samples from GS were clustered with the AWSB samples from AW for P cycling genes (S5 Fig). For all other genes, all GS Gotjawal samples were clustered together with JHDK samples

### Factors controlling soil microbial communities

**Fig 3:**
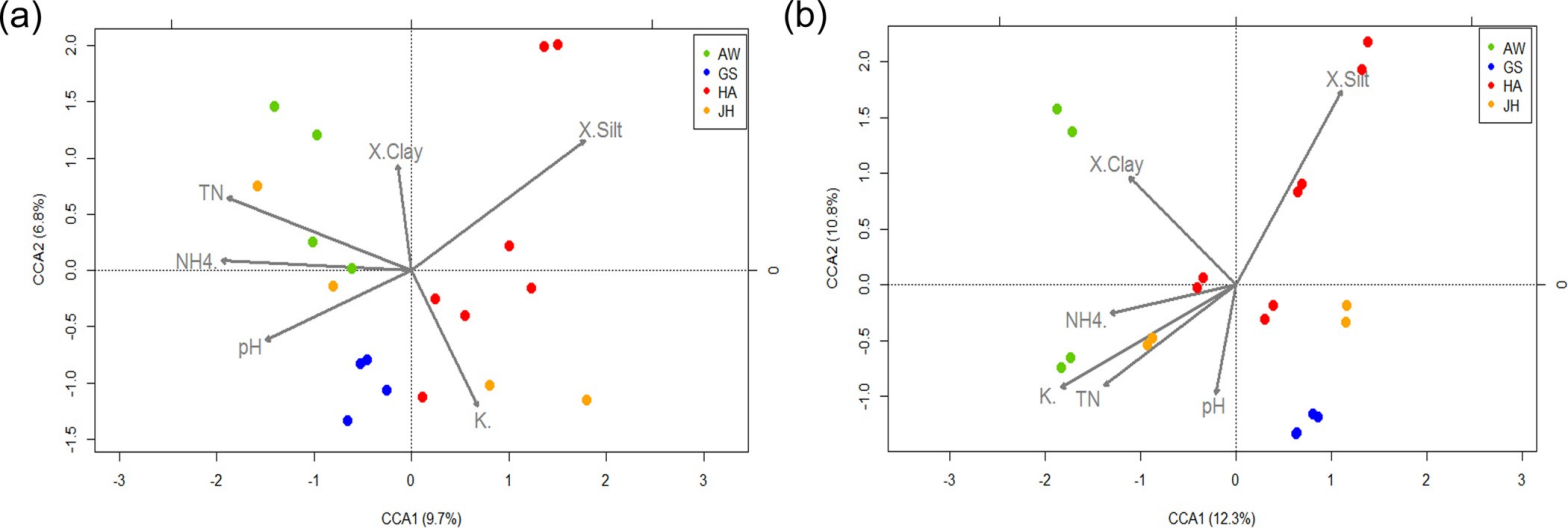
Canonical correspondence analysis for 16S rRNA gene sequence (a) and GeoChip functional gene data (b).

Bacterial communities defined by 16S rRNA genes were fitted along gradients of nitrate (deviance explained = 61.9%, *p* = 0.002) and % silt (deviance explained = 60.5%, *p* = 0.004) by a generalized additive model (GAM) (Fig 3A and 3B). Other significant environmental factors included ammonium (72.86–288.43 mg/Kg), conductivity (1.67–8.01), total N (0.79–2.83%), CEC (55.12–106.23 cmol/kg), K-exchange capacity (94.12–601.34 mg/kg). Microbial communities defined by functional genes were fitted along gradients of K-exchange capacity (deviance explained = 23%, *p* = 0.077) (Fig 3C and 3D). C and P cycle genes were fitted with K-exchange capacity, while N cycle genes were with ammonia, and S cycle genes were with nitrate and K-exchange capacity (S4 Fig). Overall fits by GAM were much less than for bacterial communities (deviance explained = 24–40%), except for S cycle genes with 60.1% deviance explained by nitrate (*p* = 0.034). Soil characteristics profiles were most significantly correlated with P cycle genes by Procrustes test (*R* = 0.413, *p* = 0.068).

The Mantel and partial Mantel test (Table 3) revealed a significant association between composited soil characteristics and distances, and bacterial communities defined by 16S rRNA genes. However microbial communities defined by functional genes did not yield significant results. These results indicated that bacterial communities were spatially autocorrelated while functional genes were not. The overall linkage of functional genes with soil characteristics was weak. P cycling genes were most significantly associated with both the soil characteristics and distance. The Procrustes test results with NMDS ordination overall agreed with Mantel test results.

**Table 3:** Mantel and partial Mantel test results with microbial communities, soil characteristics and geographic distances.

		Soil	Distance	Partial (-dist)
**16S**	*r*_M_	0.262	0.156	0.212
	*P*	**0.012**	**0.036**	**0.027**
**All**	*r*_M_	0.067	0.081	0.032
	*P*	0.186	0.153	0.325
**C**	*r*_M_	0.080	0.078	0.049
	*P*	0.175	0.145	0.279
**N**	*r*_M_	0.047	0.081	0.010
	*P*	0.258	0.155	0.429
**P**	*r*_M_	0.105	0.115	0.058
	*P*	0.105	0.069	0.241
**S**	*r*_M_	0.056	0.075	0.022
	*P*	0.209	0.144	0.364

Some soil measurements were redundant based on their collinearity, and thus were removed in building the constrained correspondence analysis (CCA) models. Through the iterative process, total N, ammonia, nitrate, Na exchange capacity, percent clay and percent silt were selected for the CCA model with bacterial communities (Fig 3A). HA samples were correlated with soil texture (HACS, HAJJ, and HASY). AW and JH samples were correlated with soil N (AWSB, AWNKM, and JHKR), while Na exchange capacity was correlated with some JH and HA samples (JHDK and HADNR). Cation exchange capacity, pH, total N, ammonia, K exchange capacity, percent clay, and percent silt were correlated with the soil microbial communities of all nutrient cycling genes. HA and AW samples were correlated with either soil texture (HASY, HACS, and AWSB) or soil nutrients (HAJJ and AWNKM). Soil nutrients were important correlating factors of JHKR samples, while pH was important for GS samples. The overall patterns for general microbial communities and functional gene categories were generally similar (Fig 3B and S6 Fig). All of the CCA models were very significant with the selected constraining soil characteristics variables (*p* < 0.001). Again, the CCA model with P cycling genes was the most unique among nutrient cycling genes, because all HA, GSDG, and JHDK samples were explained best by soil texture. AWNKM and JHKR samples were better explained by percent clay and P_2_O_5_ concentration.

### P cycling genes

In terms of the P cycling gene community based on GeoChip analysis, a *ppx* (exopolyphosphatase) gene was primarily detected in the AWNKM, AWSB, HADNR, GSBY, and GSDG soil samples. In contrast, a gene encoding *phytase* (hydrolyzing phytate to release inorganic phosphate) was primarily detected in the HACS soil samples. The *ppx* gene was not detected the HAJJ soil samples, while the *phytase* gene was not detected the HAJJ and GSBY soil samples. A *ppx* gene was detected in the AWSB, HAJJ, GSBY, and GSDG soil samples. Low detection of a *ppk* (polyphosphate kinase) gene was observed in all Gotjawal soil samples. The *phytase* gene produced no signal in AWSB, whereas *ppx* and *ppk* genes produced strong signals in this sample. In HAJJ, *ppx* and *phytase* genes were detected very strongly. Phosphorus cycling genes significantly contributed to site-specific characteristics.

Available phosphorus content was very high, but varied with Gotjawal region with values of 231, 152, 96, and 55 mg/Kg in AWNKM, HADNR, HAJJ, and JHKR, respectively. In comparison, iron and aluminum concentrations were 5640 mg/Kg and 3410 mg/Kg in AWNKM, 9790 and 6750 mg/Kg in HADNR, 18840 and 11750 mg/Kg in HAJJ, and 25540 and 21100 mg/Kg in JHKR (Table 1).

### 16S rRNA gene sequence analysis and indicator species analysis (ISA)

From the MiSeq sequence data, we examined a total of 140,716 reads representing 20 phyla from Gotjawal soils. The mean read count per sample was 7,036, with 4,898 to 8,487 reads. The mean OTU was 777 (range: 456 to 892), while the mean Shannon index was 7.857 (range: 6.8336 to 8.1935). Rarefaction curves were obtained from 4,898~ 8,487 sequences (S7 Fig and S3 Table), with most samples approaching a plateau. HACS2 was exceptional with very low OTU counts, so it was removed in the subsequent analyses. Representative sequences of the 20 soil samples were affiliated with *Proteobacteria* (27.9%), *Actinobacteria* (17.7%), *Verrucomicrobia* (14.5%), *Planctomycetes* (9.8%), *Bacteroidetes* (8.9%), *Acidobacteria* (9.6%), and *Chloroflexi* (2.2%) (S8 Fig and S4 Table). The relative abundances of these phyla were consistent across samples (S8 Fig).

**Fig 4:**
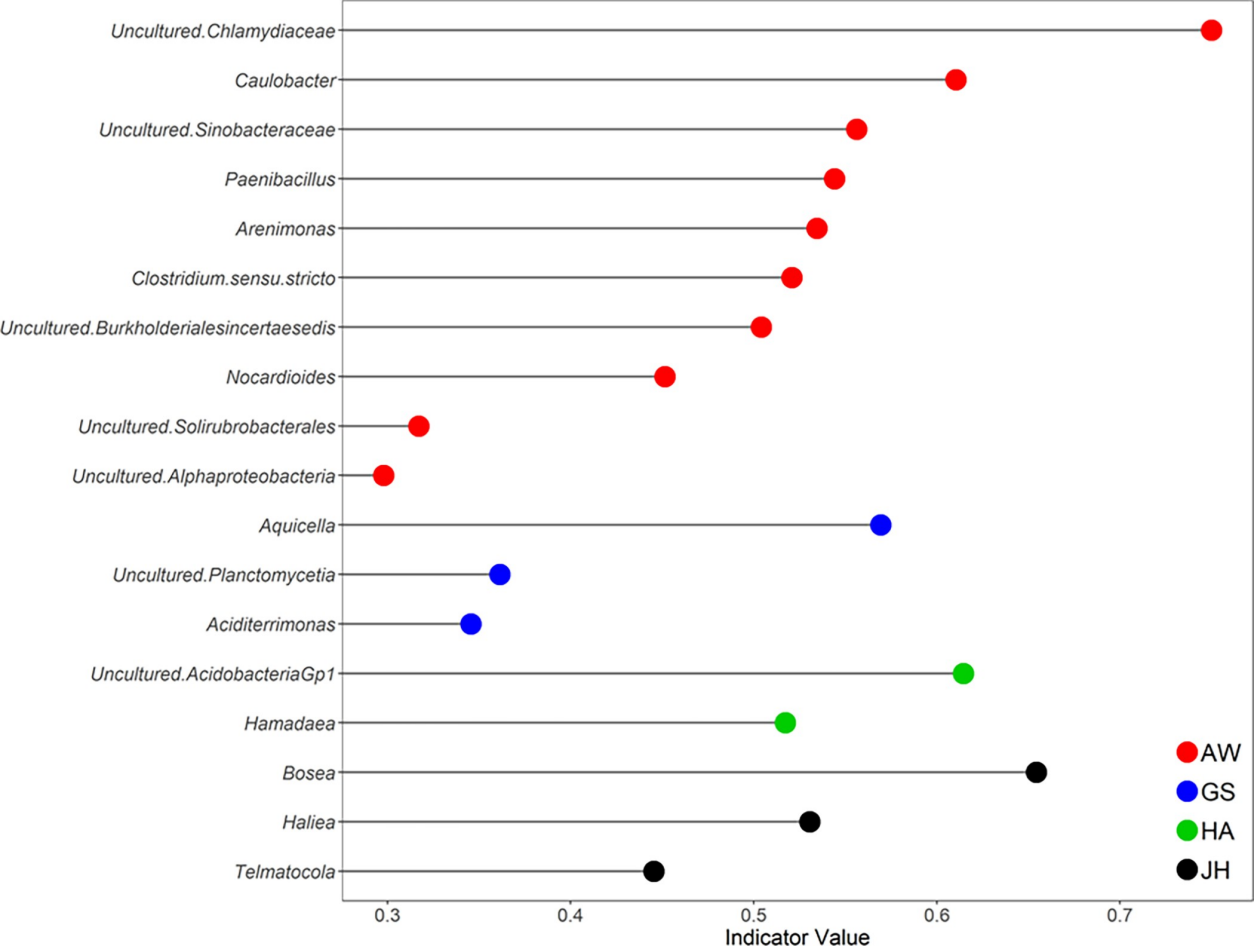
Indicator species analysis (ISA) of 16S rRNA OTUs at the genus level. The most characteristic species of the microbial community in four Gotjawal areas were identified through the indicator value using ISA.

Using indicator species analysis (ISA), we found indicator species in sites, which were represented by following taxa: uncultured *Chlamydiaceae*, *Caulobacter*, uncultured *Sinobacteraceae*, *Paenibacillus*, *Arenimonas*, *Clostridium* sensu.stricto, uncultured *Burkholderiales* incertae sedis, *Nocardioides*, uncultured *Solirubrobacterales*, and uncultured *Alphaproteobacteria* in Aewol (AW), *Aquicella*, uncultured *Planctomycetia*, and *Aciditerrimonas* in Gujwa-Seongsan (GS), uncultured *Acidobacteria* Gp1, and Hamadaea in Hankyeiong-Andeok (HA), and *Bosea*, *Haliea*, and *Telmatocola* in Jocheon-Hamdeok (JH) Gotjawal (Fig 4).

## Discussion

Microbial community structure and functional potential in the Gotjawal soils was investigated using 16S rRNA gene sequencing and GeoChip hybridization. The results demonstrated that each of four Gotjawal regions contained distinctly different soil microbial communities as defined by 16S rRNA genes and nutrient cycle functional genes (Table 2). The microbial communities based on functional gene category were similar in structure, and had similar associations with environmental factors. However, P cycling genes were the most distinctive gene category, possibly due to the different geological features and vegetation of the Gotjawal areas (S2 Table). However, further careful analysis is required to support this result.

JHKR, AWNKM, and HAJJ were closely located in NMDS ordination space (S4 Fig) and were grouped in the same cluster in HCA (S5 Fig) based on functional gene data, but they were not clustered with16S rRNA gene data.

Composite soil characteristics were consistent with the 16S rRNA gene data, since clustering of three locations was not observed (S2 Fig). Both JHKR and AWNKM have many hummock and hollows in two Gotjawal regions, secondary deciduous broad-leaved forests, and temperate climates. In comparison, HAJJ was characterized by lava flows transitioning from pahoehoe to aa, collapsed trenches, a collapsing of lava tubes, an evergreen broad-leaved forest, and a subtropical climate. JHKR, AWNKM, and HAJJ were humid with subsidence, along with extensive cattle grazing, which should have greater impact on the functional potential of their soil microbial communities.

ANOSIM and PERMANOVA were used to test whether several groups of communities are significantly different from each other by comparing between-group and within-group dissimilarity[21, 22]. The Mantel tests are a non-parametric multivariate correlation analysis between two dissimilarity matrices. When one of the matrices is a distance matrix, it tests the overall spatial autocorrelation of the other matrix[23]. Soil characteristics were spatially autocorrelated (*r*_M_ = 0.476, *p* = 0.001), but were not distinctive among the Gotjawal regions that they represented. In contrast, the microbial communities defined by functional genes were quite distinctive among the Gotjawal regions, but spatial autocorrelation was weak. The spatial distribution of the 4 Gotjawal regions is quite distinctive around Jeju Island; however, the distance matrix of the 20 sampling locations did not perfectly match the spatial distribution of total Gotjawal area. This discrepancy occurred because some inter-Gotjawal locations were as close to one another as other intra-Gotjawal locations.

16S rRNA gene sequences and functional gene hybridization results represent defined microbial communities based on taxonomic markers and functional potential, respectively. The direct comparison between them based on NMDS ordination (Procrustes test, *t* = 0.43, *p* = 0.07) and dissimilarity matrix (Mantel test, *r*_M_ = 0.22, *p* = 0.02) indicate marginal similarity between them. Hierarchical clustering and CCA models also showed noticeable distinctiveness between them. All of these results together may indicate a degree of decoupling between structure and function[24, 25], however there were some shared but non-identical responses. This pattern may mean different results per different response units between specific functional traits and entire functional gene profiles. Another noticeable feature is insignificant association between functional gene results with soil characteristics[26], while 16S rRNA gene results were well correlated with soil characteristics.

For the diversity measure, we added effective diversity along with conventional diversity index for the Shannon index (*H*’). The Shannon index is non-linear so quantitative comparison is not appropriate. Effective diversity is the expected richness to generate a specified diversity index overcoming the limitation of the Shannon index[27]. Although there was no significant difference among Gotjawal forests, functional genes tended to have lowest diversity in GS forest and 16S gene sequence data had lowest diversity in HA forest, again indicating a weak link between community structure and functional potential.

Out of the 4 nutrient cycling gene categories (C, N, P, S), P cycling genes were the most unique, with the community functional potential composition differing from the other 3 categories and better association with composite soil parameters. Available phosphorus content was very high, with proportions similar to those of iron and aluminum (Table 1). Out of all the P cycling genes, *phytase* and *ppx* were strongly detected; the *ppk* gene produced a weak signal in all Gotjawal soils. Genes for both exopolyphosphatase (*ppx*) and polypshophate kinase (*ppk*) are on polyphosphate operon[28–30] and responsible for the utilization of polyphosphate. Phytase catalyzes the hydrolysis of phytate, which is abundant in plants[31] and often most active in fungi[32] which may be the case for the Gotjawal forest soil. Generally, inorganic phosphorus (P_i_) comprise 35 ~ 70% in total soil P, and between 30% and 65% of total P is present as organic P (P_o_)[33]. Phytic acid, an inositol phosphate, represents the dominant form of organic P in soil[34, 35]. The phytate in Gotjawal forest soil could be degraded by phytase, also known as a myo-inositol phosphatase, with four distinctly different classes known[32]. Future assessments of myo-inositol phosphate and myo-inositol phosphatases will be used to develop more detailed models of P cycling and to understand differences among Gotjawal sites.

In conclusion, our study investigated the microbial community structure and functional potential in Gotjawal soils using 16S rRNA gene sequencing and GeoChip. We showed that each Gotjawal region contained noticeably different soil microbial communities, despite soil characteristics that were not clearly distinctive. The taxonomic level composition and diversity, however, of those microbial communities were not very distinct. The indicator species of the microbial community in four Gotjawal regions were identified differently as uncultured *Chlamydiaceae*, *Caulobacter*, uncultured *Sinobacteraceae*, *Paenibacillus*, *Arenimonas*, *Clostridium* sensu.stricto, and uncultured *Burkholderiales* incertae sedis in AW, *Aquicella* in GS, uncultured *Acidobacteria* Gp1, and *Hamadaea* in HA, and *Bosea*, and *Haliea* in JH. In addition, we showed that P cycling genes (phytase, *ppx*, and *ppk*) are critical in these regions, and should be the focus in future studies. Our results demonstrate that the Gotjawal area supports diverse microbial communities that, in turn, support diverse plant and wildlife species, thereby contributing to the uniqueness of this primarily unexplored ecosystem that requires conservation.

## Supporting information

S1 Fig Photos of Gotjawal areas. Hankyeong-Andeok (HA), Dorneri (HADNR), Sanyang (HASY), Cheongsu (HACS), Jeoji (HAJJ); Aewol (AW), Nokome (AWNKM), Sangbu (AWSB); Jocheon-Hamdeok (JH), Gyorae (JHKR), Dongbaekdongsan (JHDK); Gujwa-Seongsan (GS), Dunji (GSDG), Baekyagi (GSBY). The photographs by D.S.Kim.
(JPG)

S2 Fig Exploratory analysis of soil characteristics by unconstrained ordination plots using Non-metric Multidimensional Scaling (NMDS) with ellipses determined by the standard deviation of point scores (a) and hierarchical cluster analysis (HCA) using complete linkage agglomeration method with Euclidean distance measure (b).
(TIF)

S3 Fig Shannon index (*H*’) (a), and effective number of Shannon index (*H*’) of 16S rRNA gene, all functional genes and 4 nutrient cycle genes among 4 Gotjawal areas (b). Error bars represent one standard deviation.
(TIF)

S4 Fig **NMDS ordination with two most significant environmental gradients for nutrient cycle genes (a-h).** (a) C cycle genes. (b) C cycle genes, K- exchange capacity (deviance explained = 24.9%, *p* = 0.063). (c) N cycle genes. (d) N cycle genes, ammonia (deviance explained = 40.0%, *p* = 0.055). (e) P cycle genes. (f) P cycle genes, K- exchange capacity (deviance explained = 24.3%, *p* = 0.068). (g) S cycle genes. (h) S cycle genes, red: nitrate (60.1%, *p* = 0.034), gray: K- exchange capacity (24.7%, *p* = 0.065).
(TIF)

S5 Fig Hierarchical cluster analysis (HCA) of 16S rRNA gene (a) and nutrient cycle genes (b-f) using complete linkage agglomeration method with Bray-Curtis dissimilarity measure. Box and number indicate clustering groups by k-means partitioning.
(TIF)

S6 Fig Canonical correspondence analysis for microbial communities measured by nutrient cycle functional genes. (a) C cycle, (b) N cycle, (c) P cycle and (d) S cycle.
(TIF)

S7 Fig Rarefaction curves of Chao1 index with 16S rRNA sequence reads from MiSeq with 20 Gotjawal forest samples. (TIF)

S8 Fig Bacterial composition at the phylum level. (TIF)

S1 Table The geographic coordinates of the soil samples collection sites. (TIF)

S2 Table Geological features and vegetation in Gotjawal. (TIF)

S3 Table Community richness and diversity of Gotjawal soils. (TIF)

S4 Table Relative mean abundance of the phylogenetic groups presents in the Gotjawal soils. (TIF)
