# Correction: Microbial community structure and functional potential of lava-formed Gotjawal soils in Jeju, Korea

**DOI:** 10.1371/journal.pone.0211435

**Published:** 2019-01-24

**Authors:** Jong-Shik Kim, Dae-Shin Kim, Keun Chul Lee, Jung-Sook Lee, Gary M. King, Sanghoon Kang

S1 File is omitted from the list of Supporting Information. It can be viewed below.

There are errors in Table 1. In column HACS-1, row Fe, the value should read: 17127.67. In column HACS-1, row Al the value should read: 9505.00 Please see the corrected Table 1 here.

**Table 1:** Chemical properties of Gotjawal soil samples.

Gotjawal		HACS-1	HACS-2	HADNR-1	HADNR-2	HASY-5	HASY-6	HAJJ-1	HAJJ-2	AWNKM-1	AWNKM-2	AWSB-1	AWSB-2	JHKR-1	JHKR-2	JHDK-1	JHDK-2	GSBY-1	GSBY-2	GSDG-1	GSDG-2
**pH**		5.5	4.0	5.3	6.2	4.2	4.5	5.3	4.6	5.0	5.3	5.1	5.5	4.5	4.5	5.2	4.5	5.5	5.6	5.4	5.7
**EC**	dS/m	2.69	2.62	5.06	3.83	1.67	2.33	2.05	1.88	7.41	8.01	3.61	4.03	2.80	4.09	1.87	1.69	3.72	3.15	3.26	3.59
**OM**	%	44	46	50	45	25	30	43	34	66	66	36	69	33	35	29	31	44	43	42	46
**TOC**	%	25.25	26.57	29.24	25.91	14.61	17.28	25.15	19.85	38.24	38.30	21.10	40.19	19.17	20.14	16.83	18.04	25.44	25.09	24.35	26.79
**TN**	%	1.71	2.31	2.39	2.15	0.79	1.30	2.32	1.52	2.46	2.64	1.71	2.83	1.49	1.83	1.36	1.33	2.03	1.97	2.60	1.98
**NH**_**4**_**(+)**	(mg/kg)	72.860	70.595	113.961	128.080	174.471	93.790	110.935	115.978	150.267	124.046	259.186	164.386	255.151	288.432	91.774	80.680	156.318	154.301	168.420	155.309
**NO**_**3**_**(-)**	(mg/kg)	132	573.17	330.004	434.217	204.950	149.371	243.160	166.740	500.216	406.428	389.059	628.746	191.057	409.900	114.651	66.017	357.793	361.270	382.110	455.058
**CEC**	(cmol/kg)	66.05	75.19	95.88	86.28	55.74	62.88	67.74	55.12	104.35	106.23	76.95	101.02	63.62	63.43	59.31	56.80	94.51	94.51	72.29	74.28
**Exch. Cation (mg/kg)**	Ca	3476.3	1100.56	8569.35	8320.66	97.53	721.33	5616.33	872.46	8172.99	9914.45	3793.39	8897.49	729.57	1745.03	2557.95	568.87	5997.16	5859.38	4983.58	6283.64
	Mg	868.86	400.75	1723.10	1273.95	299.07	196.04	1089.31	557.90	1511.22	1688.29	646.63	1446.84	167.56	316.82	813.11	337.37	866.75	838.50	618.30	866.29
	K	179.43	139.79	500.60	418.60	96.60	94.12	263.66	238.21	601.34	511.97	369.26	446.59	232.30	258.42	184.85	203.06	315.88	284.01	265.84	341.37
	Na	82.63	39.04	54.63	71.58	43.60	18.27	65.83	55.74	80.21	75.87	23.17	63.81	30.26	35.76	84.13	87.02	44.38	44.46	58.06	56.39
**Aval. P**_**2**_**O**_**5**_		79.98	70.13	203.54	99.59	33.47	112.47	135.88	57.11	250.72	214.64	84.17	119.55	37.86	59.03	49.09	61.15	91.39	97.50	109.35	68.41
**soil texture**		loam	clay	clay loam	sandy loam	silt loam	silt clay loam	clay loam	clay loam	clay	sandy clay	sandy clay loam	silty clay	sandy loam	sandy loam	loam	loam	sandy loam	sandy loam	sandy clay loam	sandy loam
**sand**		38.87	11.47	32.26	66.82	1.99	2.78	29.62	20.07	39.57	51.87	64.19	3.65	66.97	56.11	46.92	32.46	61.13	62.13	61.52	74.54
**silt**	%	35.28	37.79	36.22	20.89	77.90	69.51	42.73	50.41	19.84	11.19	9.93	53.26	19.66	25.80	33.42	45.81	23.16	24.60	17.99	14.64
**clay**		25.86	50.74	31.52	12.29	20.11	27.71	27.65	29.52	40.59	36.94	25.87	43.10	13.37	18.09	19.66	21.74	15.71	13.26	20.48	10.81
**Heavy Metal**	**(mg/kg)**																				
**Fe**		#####	12421.00	4946.50	14637.67	38434.33	19017.67	14394.33	23287.67	6794.75	4482.25	15551.00	5846.50	35967.67	22397.67	28677.7	25001.00	19717.67	23231.00	24461.00	19381.00
**Mn**		438.60	200.60	221.77	540.60	236.67	209.60	634.60	424.27	415.85	534.40	869.60	427.93	429.93	330.20	440.93	482.27	763.60	935.93	790.60	689.93
**Si**		241.03	235.97	172.70	361.90	217.03	455.57	331.23	274.73	378.25	441.35	674.90	179.33	162.00	657.23	248.23	350.57	451.90	1325.23	603.23	629.57
**Al**		#####	4801.67	3002.00	10488.33	14181.67	8758.33	9341.67	14151.67	3928.50	2887.00	6645.00	3258.67	24265.00	17935.00	14381.7	17355.00	15341.67	18751.67	13348.33	15198.33
**V**		36.73	28.07	9.03	28.17	91.47	44.47	27.53	48.67	12.45	7.70	30.27	10.90	66.00	35.17	64.57	49.53	39.70	47.53	53.80	41.50
**Cd**		1.23	0.87	0.60	1.23	2.30	1.13	1.27	1.50	1.05	1.15	1.37	1.13	2.57	1.77	1.93	1.80	1.63	1.87	2.00	1.57
**Cu**		10.77	8.97	11.60	19.33	4.37	10.13	15.27	13.73	19.20	21.75	17.63	18.27	20.47	15.13	11.97	15.37	23.73	29.20	26.27	19.07
**Pb**		16.03	19.27	16.30	37.43	21.30	19.87	26.13	19.83	40.90	37.75	78.43	36.83	23.37	25.30	23.93	22.17	29.43	32.00	32.37	22.83
**Ni**		12.50	7.80	7.13	17.27	13.60	8.17	12.80	20.50	8.85	9.10	10.00	7.87	20.70	13.93	12.33	18.03	16.83	20.23	19.90	24.77

## Supporting information

S1 File Site description and Jeju Island. (DOC)
