# Supplementary figures and images for: Microbial community structure and functional potential of lava-formed Gotjawal soils in Jeju, Korea
Source: PLoS One. 2018 Oct 12;13(10):e0204761. doi: 10.1371/journal.pone.0204761 (PMC6193574; doi:10.1371/journal.pone.0204761)

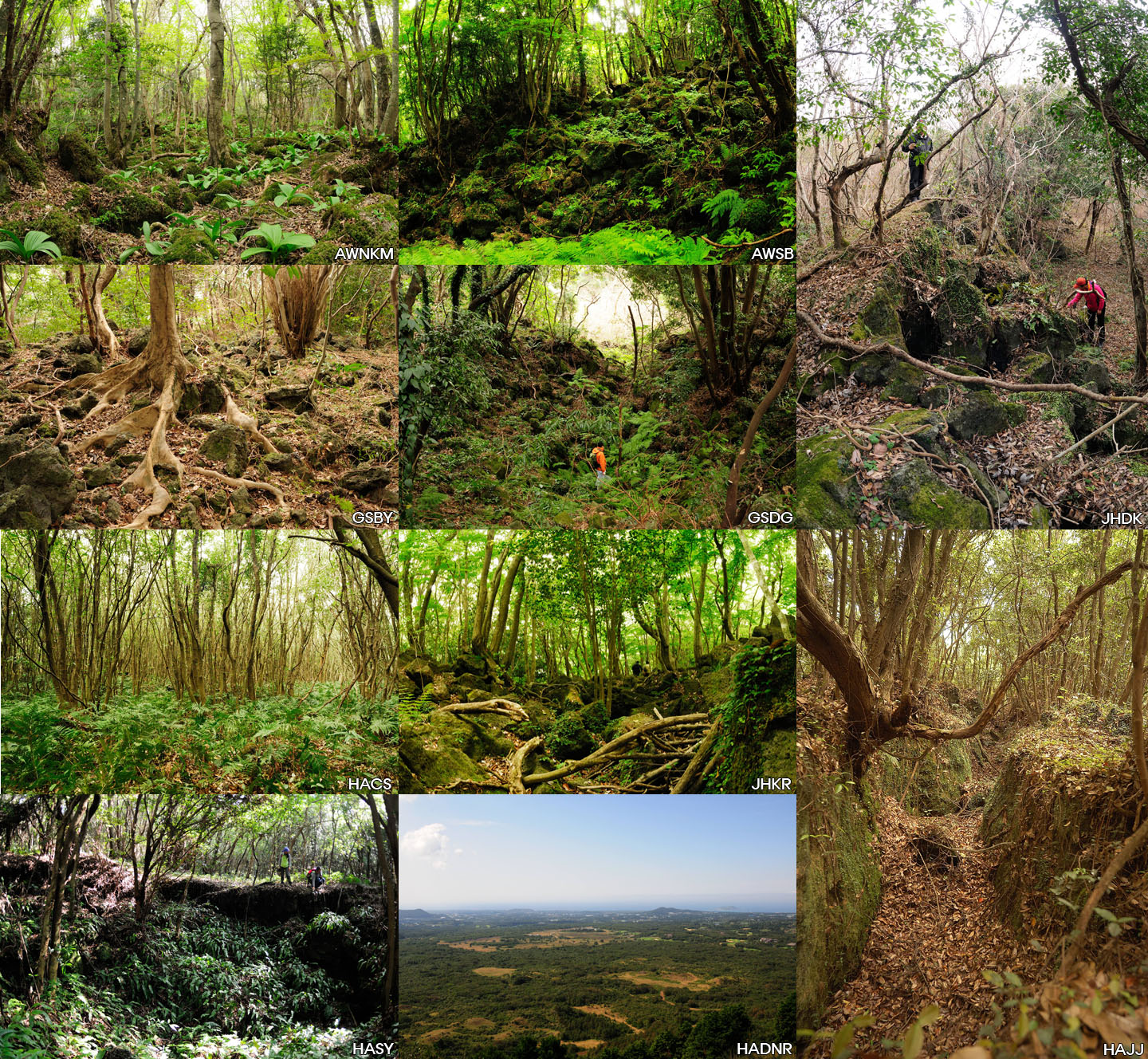

Supplement: S1 Fig — Hankyeong-Andeok (HA), Dorneri (HADNR), Sanyang (HASY), Cheongsu (HACS), Jeoji (HAJJ); Aewol (AW), Nokome (AWNKM), Sangbu (AWSB); Jocheon-Hamdeok (JH), Gyorae (JHKR), Dongbaekdongsan (JHDK); Gujwa-Seongsan (GS), Dunji (GSDG), Baekyagi (GSBY). The photographs by D.S.Kim. (JPG) [file pone.0204761.s001.jpg]

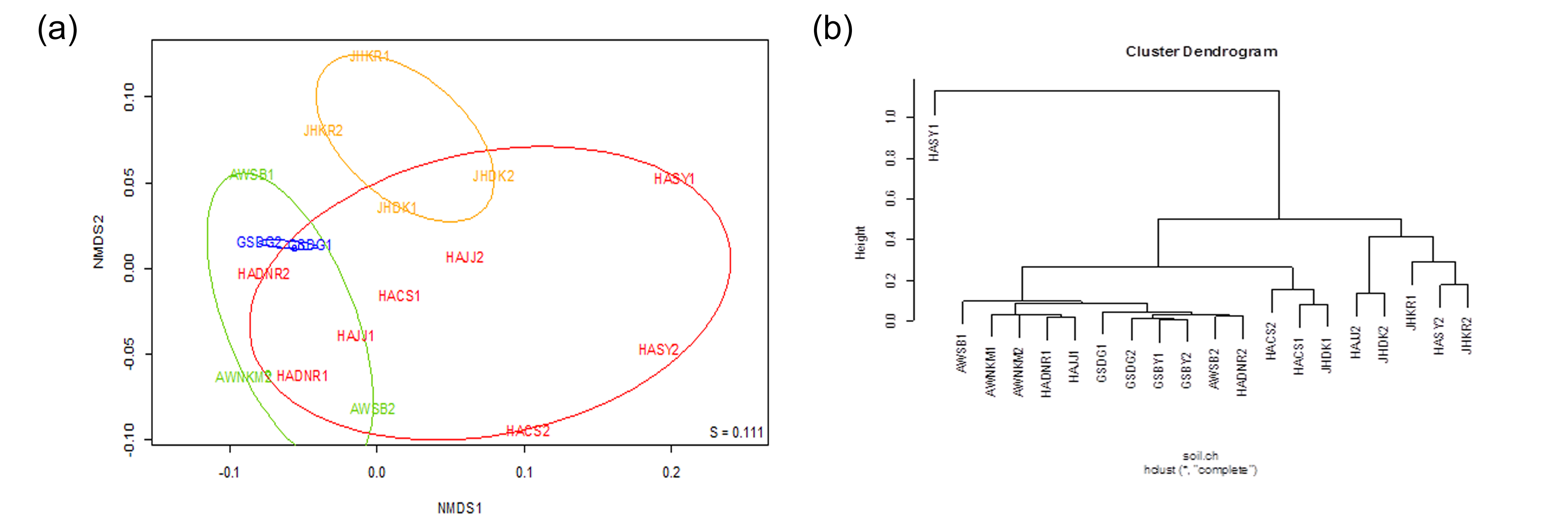

Supplement: S2 Fig — Exploratory analysis of soil characteristics by unconstrained ordination plots using Non-metric Multidimensional Scaling (NMDS) with ellipses determined by the standard deviation of point scores (a) and hierarchical cluster analysis (HCA) using complete linkage agglomeration method with Euclidean distance measure (b). (TIF) [file pone.0204761.s002.tif]

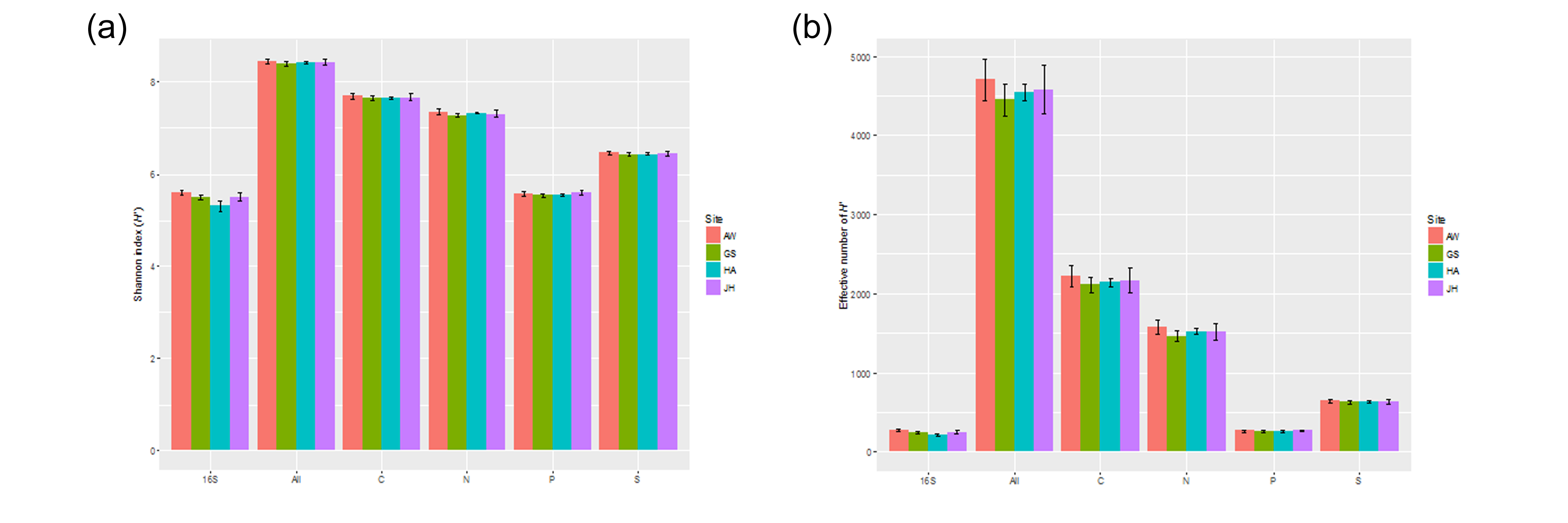

Supplement: S3 Fig — Shannon index (H’) (a), and effective number of Shannon index (H’) of 16S rRNA gene, all functional genes and 4 nutrient cycle genes among 4 Gotjawal areas (b). Error bars represent one standard deviation. (TIF) [file pone.0204761.s003.tif]

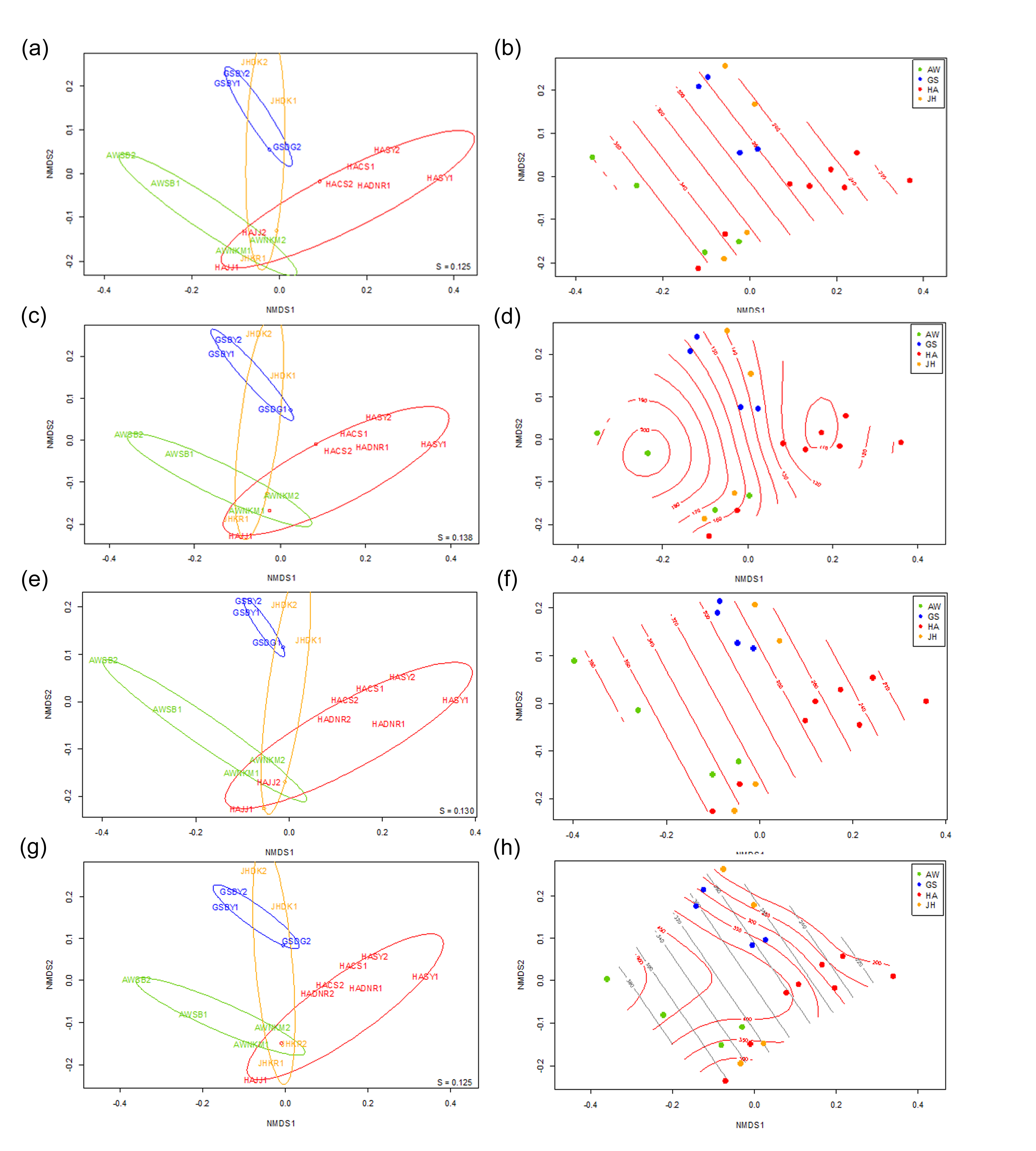

Supplement: S4 Fig — NMDS ordination with two most significant environmental gradients for nutrient cycle genes (a-h). (a) C cycle genes. (b) C cycle genes, K- exchange capacity (deviance explained = 24.9%, p = 0.063). (c) N cycle genes. (d) N cycle genes, ammonia (deviance explained = 40.0%, p = 0.055). (e) P cycle genes. (f) P cycle genes, K- exchange capacity (deviance explained = 24.3%, p = 0.068). (g) S cycle genes. (h) S cycle genes, red: nitrate (60.1%, p = 0.034), gray: K- exchange capacity (24.7%, p = 0.065). (TIF) [file pone.0204761.s004.tif]

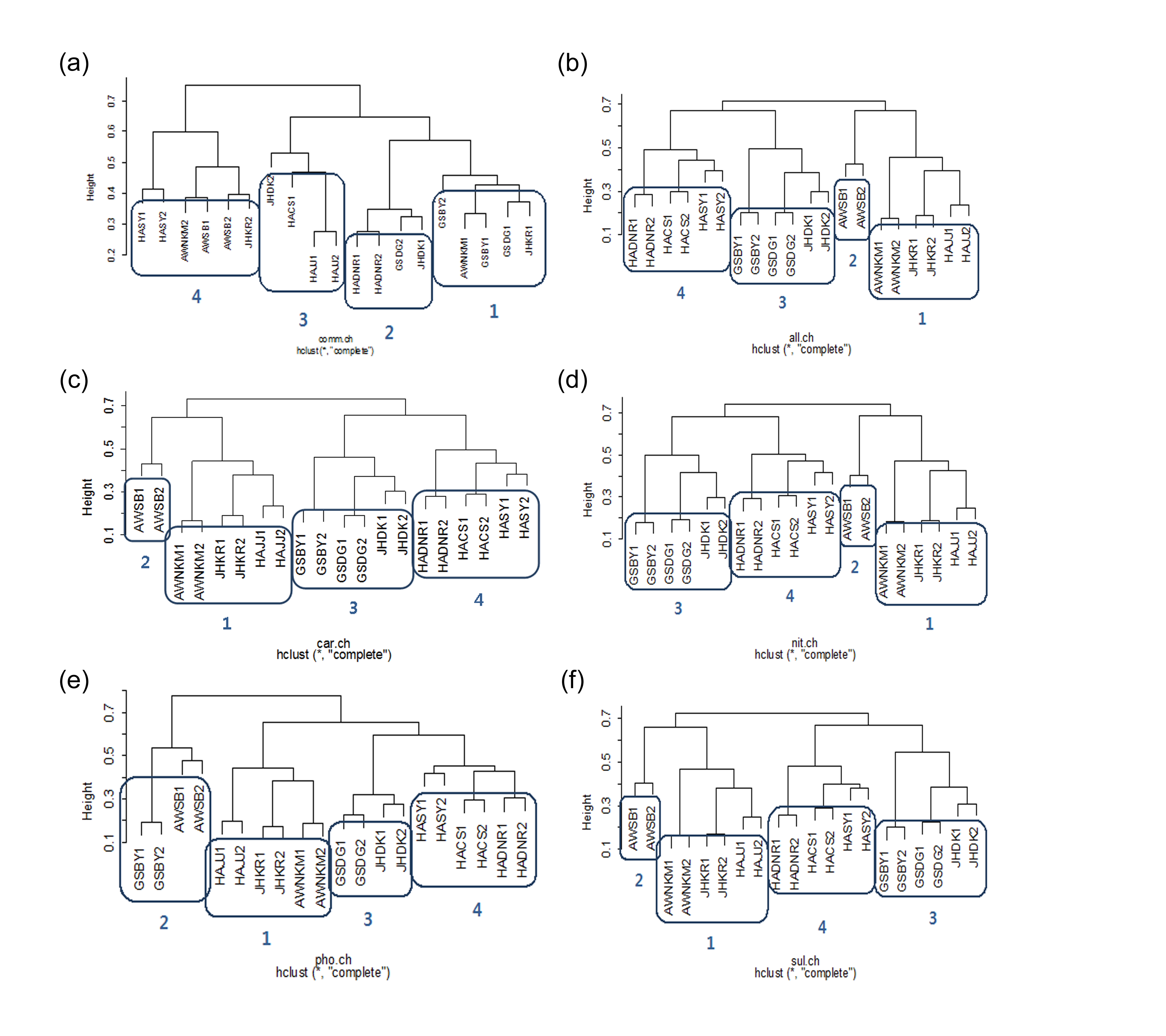

Supplement: S5 Fig — Hierarchical cluster analysis (HCA) of 16S rRNA gene (a) and nutrient cycle genes (b-f) using complete linkage agglomeration method with Bray-Curtis dissimilarity measure. Box and number indicate clustering groups by k-means partitioning. (TIF) [file pone.0204761.s005.tif]

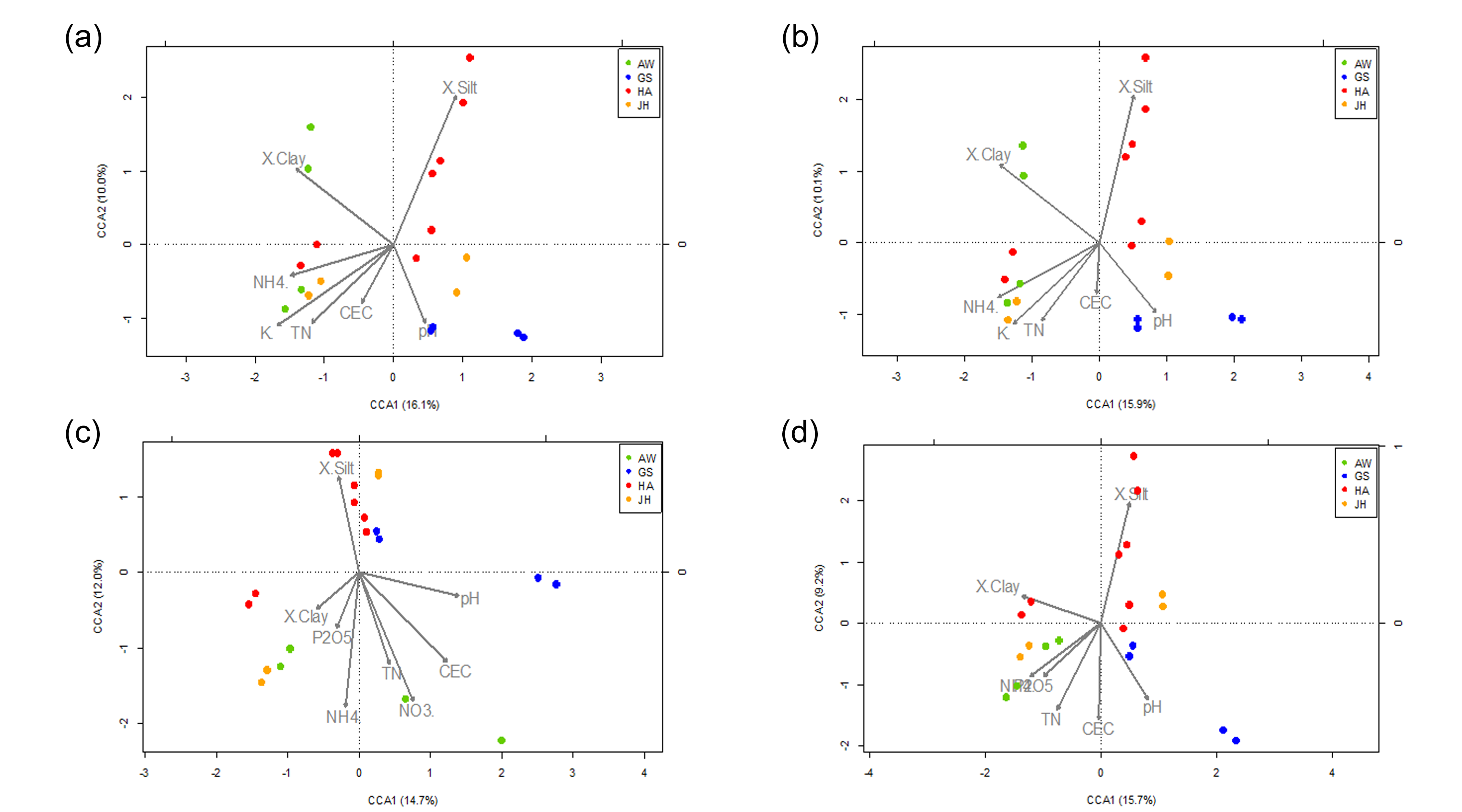

Supplement: S6 Fig — (a) C cycle, (b) N cycle, (c) P cycle and (d) S cycle. (TIF) [file pone.0204761.s006.tif]

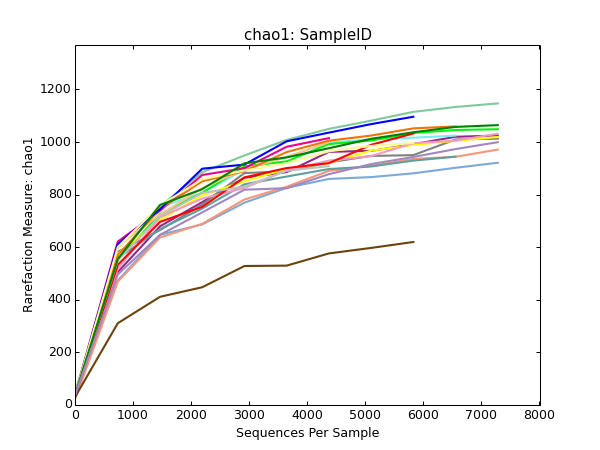

Supplement: S7 Fig — (TIF) [file pone.0204761.s007.tif]

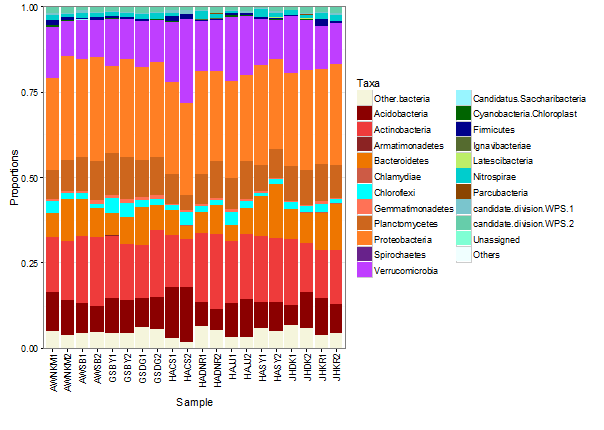

Supplement: S8 Fig — (TIF) [file pone.0204761.s008.tif]

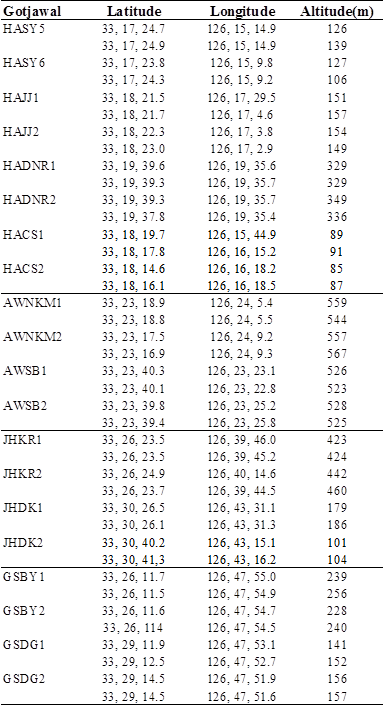

Supplement: S1 Table — (TIF) [file pone.0204761.s009.tif]

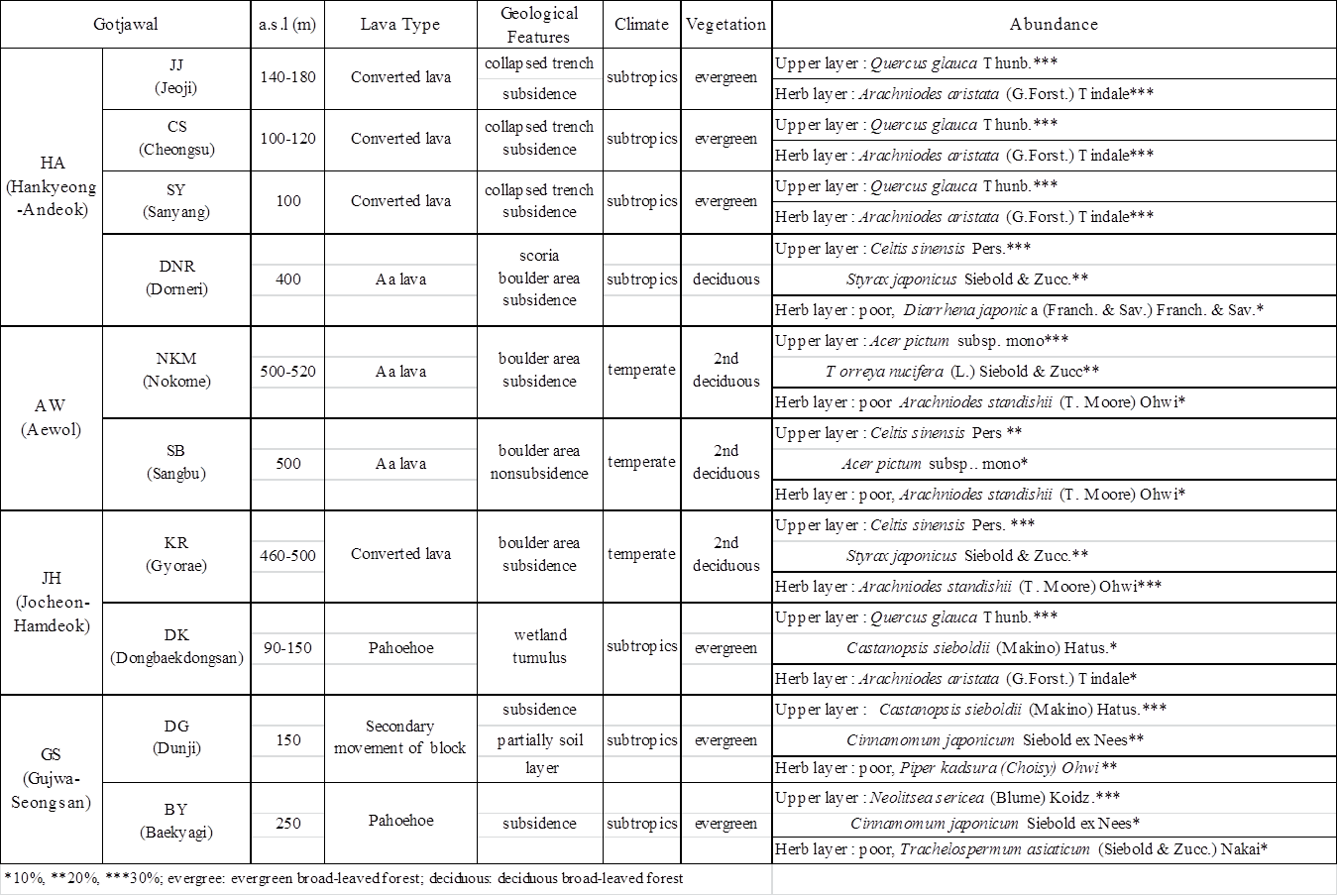

Supplement: S2 Table — (TIF) [file pone.0204761.s010.tif]

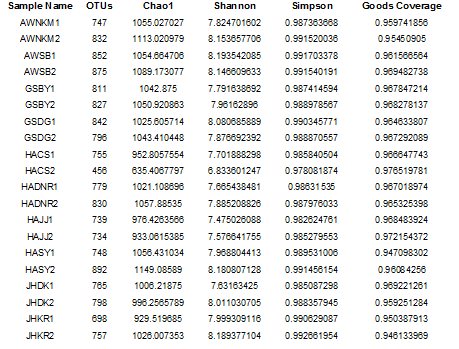

Supplement: S3 Table — (TIF) [file pone.0204761.s011.tif]

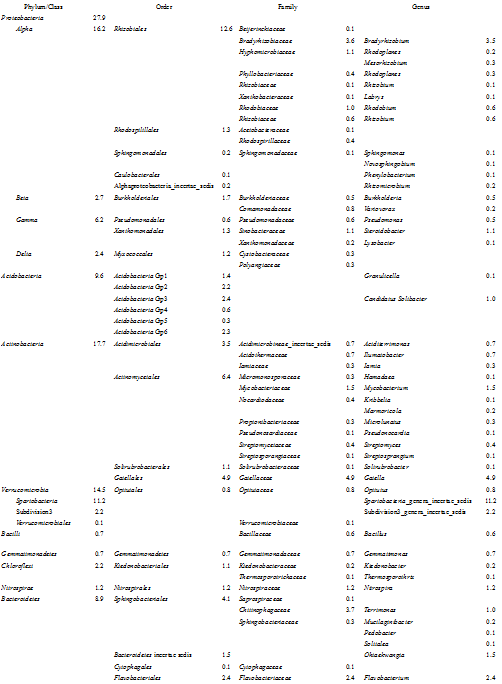

Supplement: S4 Table — (TIF) [file pone.0204761.s012.tif]
