# Supplementary material for: Correction: Microbial community structure and functional potential of lava-formed Gotjawal soils in Jeju, Korea
Source: PLoS One. 2019 Jan 24;14(1):e0211435. doi: 10.1371/journal.pone.0211435 (PMC6345446; doi:10.1371/journal.pone.0211435)
Supplement: S1 File — (DOC) [file pone.0211435.s001.doc]

**Site description**

Gotjawal forests are located along the east-west axis of Jeju Island, Korea. Most forests occur in inland areas at altitudes of 200 to 400 m. Gotjawal forests tend to form a zone between inhabited coastal areas and mountainous regions used for grazing livestock. Four major Gotjawal forests are spread across Jeju Island, separated by Mount Hallasan (1,950 m) in the middle of the island (Fig. 1). These 4 forests have been preserved well, specifically (1) Hankyeong-Andeok (HA), HA Gotjawal is located in the southwest region of Jeju at 70–350 m above sea level (a.s.l.). This region was formed by Pahoehoe Lava, except for the HA Dorneri (HADNR) Gotjawal, which was formed from ‘A’a Lava. The dominant tree species is ring-cupped Oak (*Quercus glauca*Thunb*.*) in subtropics evergreen broad-leaved forests. In comparison, the HADNR Gotjawal is a secondary deciduous broad-leaved forest. Japanese Hackberry (*C. sinensis* Pers*.*) and *Styrax japonicus* Siebold ex Nees dominate the temperate broad-leaved forest at 400 m a.s.l. However, the HA Jeoji (HAJJ), Cheongsu (CS), and Sanyang (SY) Gotjawal are collapsed trenches. (2) Aewol(AW), AW Nokome (AWNKM), and AW Sangbu (AWSB) Gotjawals are located on ‘A’a Lava in the northwest region of Jeju, at 400–600 m a.s.l. These two Gotjawals are characterized by subsidence process. Following lava flow, a cave formed, whose surface collapsed, creating a lava subsidence (S1 Fig). These Gotjawals contain secondary deciduous broad-leaved forests, dominated by Mono maple (*Acer pictum* subsp. *mono*) and Japanese Hackberry (*C. sinensis*) in a temperate climate. (3) Jocheon-Hamdeok (JH), JH Dongbaekdongsan (JHDK) is located in the northeast region of Jeju at 90–150 m a.s.l. This area is primarily formed from mixtures of ’A’a Lava and Pahoehoe Lava rocks, which allow rainwater to retain under Gotjawal forests and contribute to a higher rate of groundwater recharge as one of main groundwater sources for Jeju people. Ring-cupped Oak (*Q. glauca*) and *Castanopsis sieboldii* are the dominant vegetation, forming a subtropics evergreen broad-leaved forest. This region is characterized as domed-shaped inflated tumulus formed on cooling Pahoehoe Lava surface, which forms among Jeju Gotjawals. The site also contains a unique area of endemic *Mankyua jejuense*, supporting a variety of recently discovered rare plant genera. JH Gyorae (JHKR) is located in the northeast region of Jeju at 400 m a.s.l., on ‘A’a Lava. The forest has developed a unique micro-landscape, including lava subsidence from a pocket-shaped collapse to the lower level of 15 m deep and 30 to 60 m wide (S1 Fig). The vegetation in this area is mainly secondary deciduous broad-leaved trees, growing in a temperate climate. Dominant species include *Celtis sinensis* Pers, *Styrax japonicus* Siebold & Zucc, *Zelkova serrata* Thunb. Makino, and *Acer palmatum* Thunb. ex Murray (Maple). The dominant herb layer species is *Arachniodes standishii* (T. Moore) Ohwi. (4) Gujwa-Seongsan (GS), GS Dunji (GSDG), and GS Baekyagi (GSBY) are located in the eastern region of Jeju, at 150–250 m a.s.l. The site is characterized by a large subsidence formation. GSDG is formed from Pahoehoe Lava, while GSBY is formed from mixed ‘A’a Lava and Pahoehoe Lava. Dominant tree species in the forest include Sericeous Newlitse (*Neolitsea sericea*) and Japanese Cinnamon (*Cinnamomum japonicum* Siebold ex Nees), forming a subtropics evergreen broad-leaved forest. AW is highest site, with both temperate and warm-temperate vegetation. JH also has both temperate and subtropics vegetation, within which JHDK was designated as a Ramsar wetland site in 2011. The whole Gotjawal areas have fewer stream systems than non-Gotjawal areas in Jeju, whereas the areas play a substitution role of streams because the formation of Gotjawal forest on basalt flow lava rocks is not able to lead to the formation of streams. The locations of all sampling sites used in this study, with photographs, are shown in Fig. 1 and the supporting material (S1 and S2 Table and S1 Fig).

**Jeju Island**

Jeju Island is a volcanic system that has been active for about 1.8 Ma on the approximately 100-m-deep continental shelf in the southeastern Yellow Sea off the southern coast of the Korean Peninsula, with the last eruption reportedly around 1000 CE. It is located approximately 650 km behind the subduction zone\ where the Philippine Sea plate slides beneath the Eurasian plate. The island is about 70 X 30 km and consists of a broad and gently sloping lava shield and a 1950 m-high central peak with steeper flanks. Over 300 individual eruptive centers are dispersed over the surface of the island, with possibly as many centers buried by historical lava flows.

Jeju Island is located in the southernmost part of Korea (126° 08'~ 126° 58', 33° 06'~ 34° 00'), and it has a temperate climate due to turbulence in the sea. The average annual temperature varies geographically from 15.3 to 16.9℃(Seogwipo City), and the average annual precipitation varies from 1,585 mm (Jeju City) to 2,393 mm (Seogwipo City). The island contains the natural World Heritage Site Jeju Volcanic Island and Lava Tubes (Brenna et al., 2015).

Brenna M, Nemeth K, Cronin S, Sohn Y, Smith I, & Wijbrans J. Co-located monogenetic eruptions

~200 kyr apart driven by tapping vertically separated mantle source regions, Chagwido, Jeju Island,

Republic of Korea. Bulletin of Volcanology. 2015; 77: 43. Doi: 10.1007/s00445-015-0928-9.
